# Development of a Digital Video-Based Occupational Risk Assessment Method

**DOI:** 10.3389/fpubh.2021.683850

**Published:** 2021-06-10

**Authors:** Nils Ove Beese, Francisca S. Rodriguez, Jan Spilski, Thomas Lachmann

**Affiliations:** ^1^Center for Cognitive Science, Department of Cognitive and Developmental Psychology, University of Kaiserslautern, Kaiserslautern, Germany; ^2^Centro de Ciencia Cognitiva, Facultad de Lenguas y Educación, Universidad Nebrija, Madrid, Spain

**Keywords:** occupational health, risk assessment, video analysis, occupational safety, construction work

## Abstract

The development and implementation of an observational video-based risk assessment is described. Occupational risk assessment is one of the most important yet also challenging tasks for employers. Most assessment tools to date use questionnaires, expert interviews, and similar tools. Video analysis is a promising tool for risk assessment, but it needs an objective basis. A video of a plastering worker was recorded using a 360° camera. The recording was then analyzed using the developed observational matrix concerning *Work Characteristics, Work Activities* as well as potential risks. Risk factors present during the video of the work included lifting, fall from ladder, hazardous substances as well as occasionally bad posture. The worker had no or just one risk factor present during most of the time of the video recording, while only 16 s with more than one risk factor present according to the observational matrix. The paper presents a promising practical method to assess occupational risks on a case-by-case basis. It can help with the risk assessment process in companies which is required by law in some industrialized countries. The matrix in combination with video analysis is a first step toward digital observational risk assessment. It can also be the basis of an automated risk assessment process.

## Introduction

Occupational risk assessment is not only a legal requirement in many industrialized countries, but also an essential factor in ensuring worker productivity and well-being. A lot of research has been done to develop appropriate instruments to improve and adapt the work environment. However, the current technological developments and rapidly changing work conditions (1, 2) make it more difficult to do a systematic and comprehensive risk assessment (3).

Occupational risk assessment is far more than just preventing accidents or injuries. According to the Guidance on risk assessment at work (4), it involves hazard identification, identification of those at potential risk from said hazards, risk estimation, possibility of risk elimination, and a judgement on further measures for prevention. This also includes exposure to hazardous substances. The Technical Rules for Hazardous Substances [TRGS, (5)], for instance, reflect the state of the art in Germany for occupational medicine and industrial hygiene and other secured scientific findings for work activities involving hazardous substances, their classification, and labeling. Within the scope of its application, the TRGS specifies requirements of the Ordinance on Hazardous Substances [GefStoffV, (6)] and the Ordinance on Occupational Medical Precautions [ArbMedVV, (7)]. In addition to corrosive or other toxic substances, the handling of dusts, for example, as they occur during drilling and grinding, is also regulated and must be taken into account as part of the risk assessment. In addition, other physical noxae, such as noise, must also be taken into account in the context of risk assessments, as well as physical (e.g., occupational health standards such as ISO 11226) and psychological requirements.

Thus, in a complete risk assessment, factors such as hazardous work situations, recommended exposure levels, and risk exposures interventions are identified (8). To gather all relevant information, researchers recommend employers to use a combination of questionnaires and observation (9). Such self-reported questionnaires are, for instance, the Management Standards Indicator Tool [MSIT, (10)] or the Copenhagen Psychosocial Questionnaire [COPSOQ, (11)]. The advantage of questionnaires is that they cover a great range of social and psychological risk factors. The disadvantage is that they are subjective and important information might be missed.

## Context

Observational risk assessment methods have the advantage that an independent observer monitors the work and takes notes on potentially risky situations. However, both the observation and the subsequent analysis of the material are very time consuming. Attempting to reduce the time required for a complete risk assessment is, unfortunately, a dangerous option: parts of the work steps would have to be omitted and thus potential risks could be overlooked.

Multiple systematic observational tools used to assess factors such as the intensity, uncertainty, organizational problems, environmental influences, and unbalanced loads associated with a task are currently available. A study by Nadri et al. (12) compared three of the existing observational risk assessment tools [the REBA computer-assisted dialogue process for the evaluation and design of work activities allowing for occupational safety and health, the Quick Exposure Check (QEC) ergonomic risk assessment technique, and the Nordic Musculoskeletal Questionnaire (NMQ)] and found that none of these assessments correlated well to actual musculoskeletal disorders. We therefore propose that more objective observational methods need to be developed in order to achieve more reliable risk assessments.

One option for employers to increase the reliability of risk assessment is the use of specifically trained experts to administer questionnaires and observe the workplace. Data collected by such experts are more reliable than observations made by people with less training, even when using the same materials. Further, experts can judge the observed tasks in the context of the entire work process and see aspects that other people may miss. A study by Offermans et al. (13) evaluated the reliability of expert exposure estimates (13). Interestingly, the case-by-case expert assessments came with the lowest prevalence of occupational risks (13). It is unclear whether non-experts overestimate or if experts underestimate risks. Relying on expert assessments, especially when summarized in job-exposure matrices, might lead to underestimation of risks because each worksite is different (14). Jones and Kumar (14) collected data in sawmill facilities using the Rapid Upper Limb Assessment (RULA), the Rapid Entire Body Assessment [REBA, (15)], the American Conference of Governmental Industrial Hygienists Threshold Limit Value (ACGIH TLV), the Strain Index (SI), and the concise exposure index [OCRA, (14)] and found substantial differences between the sawmill facilities. Accordingly, it seems that it is necessary to conduct a risk assessment in each worksite separately.

With questionnaires, observational assessment tools, expert evaluations, and job-exposure matrices, employers have a sufficient number of tools to conduct risk assessment. However, they each come with a number of limitations that leave the risk assessment incomplete. One limitation of the current assessment methods is that a human observer cannot process the range of information needed for holistic judgement (16).

Furthermore, temporal aspects of work activities are important because the risk might be built up through a combination of factors over time. It is not easy to take all of these aspects into consideration. A new approach is therefore to use video technology to enhance risk assessment.

Several different ways of employing video cameras and recordings in the estimation of risks have been explored. Neumann et al. (17) made video recordings to estimate trunk angles and angular velocities in assembly line workers and found a good interrater reliability for velocity and excellent estimations of trunk angles. Pehkonen et al. (18) used videos to assess the musculoskeletal load in kitchen workers. They extracted posture, frequency, and duration of activity and weights handled from the recordings and concluded that it was difficult for observers to estimate weight and temporal aspects with certainty. Hernandez et al. (19) used videos for an ergonomic assessment for spacesuit training. The video recordings of four tasks were analyzed together with a motion tracking device for orientation in space. They were able to accurately extract information on the angles and the time spent in each angle from the video recordings. Heberger et al. (20) used videos for risk assessment of maintenance workers in mineral processing and coal preparation plants. They used the videos to extract information on surface type (e.g., wood, machinery), surface condition (wet, dry), presence of obstacles, gross posture, and trunk position. They concluded that it was sometimes difficult to recognize these features from the recordings and that experience in the job makes this easier. Forsman et al. (21) used video recordings to complement the QEC for ergonomics. The employees watched the videos and gave their ratings on body region of discomfort, the level of pain/discomfort, and the occurrences per time unit. In addition, an observer judged the degree of static/dynamic work and the work postures of back, shoulder-arm, wrist-hand, and neck. They concluded that the video analysis combined with the QEC added value to the overall assessment while only being slightly more time consuming. Paquet et al. (8) optimized the way the video recordings were analyzed by computerizing the analysis with the video data. They were able to successfully model trunk flexion and work cycle time in axle inspection, loading, and relay rod upsetting. A recent paper from McKinnon et al. (22) reports on a comparison of video-based and traditional assessment of physical demands analysis of 10 simulated work tasks. They analyzed the angle of the joints and the height of the hand in the room. Overall, their results suggest that the video-based posture analysis came with better ratings than the traditional assessment. Taking all of these results together, it seems that video recordings have a high usability for risk assessment. Yet, most of the work done so far focused on a few biomechanical parameters and did not include the variety of risk factors that need to be considered in a general risk assessment.

One method that offers the possibility of a much more complete risk assessment is Video Exposure Monitoring (VEM), also known as Picture Mix Exposure [PIMEX, (23, 24)]. Gressel et al. combined video recording with air monitoring instruments to measure the concentration of pollutants present during the recorded work. A review by Rosen et al. (25) showed that it is mainly used to identify reasons for hazardous exposure and potential risks and also can work as a catalyst to reduce those because it is shown to the employees in real time. Beurskens-Comuth et al. (26) also used a newer version of PIMEX in combination with several monitoring instruments to assess the amount of nanoparticles the employees were exposed to. They concluded that PIMEX in their setup is an effective method to identify peak exposure during work.

Even though video-based risk assessment seems promising, it is not yet in a state that it can be used to carry out a systematic and comprehensive risk assessment. The aim of this paper was to develop a method to accomplish that. The objective of the work presented in the following sections was to develop a method based on video analysis to assess a variety of risk factors in a general risk assessment. Specifically, with the example of construction work, we used a 360° camera that recorded strategically selected work sites. Information on occupational risk situations were then systematically extracted from the video recordings.

In this community case study, we describe the development and implementation of an observational matrix for risk assessment on a case-by-case basis that is built to be used in conjunction with videos.

## Detail

### Classification of Risk Factors

For risk assessment, we employed an approach using the manual on hazard factors by the German Federal Institute for Occupational Safety and Health (27) as well as the health report on plasterers (Arbeitsmedizinischer Dienst der BG BAU^1^; see Table 1). The rules for occupational safety and health on construction sites [RAB, (28)] reflect the state of the art with regard to safety and health on construction sites. They were drawn up by the Committee for Safety and Health on Construction Sites (ASGB). The RAB 10 contains definitions of the Ordinance on Safety and Health at Construction Sites [BaustellV, (29)].

**Table 1:** Hazard factors according to the manual on hazard factors and health report.

**Hazard factors**	**Can be analyzed with the developed matrix**	**Can be captured on video**
Heavy lifting	✓	✓
Noise	✓	✓^*^
Physically taxing work	✓	✓^*^
Vibrations of parts of the body	✓	✓
Hazardous substances	✓	✓^*^
Vibrations of the whole body	✓	✓
Forced posture	✓	✓^*^
Lighting	✓	✓^*^
Temperature	✓	✓^*^

* *Might require additional technical equipment or methods*.

The video recordings were analyzed systematically as described in the following. Relevant work aspects were described at the beginning of the video recording as *Work Characteristics* and *Work Activities* in the matrix. *Work Characteristics* are defined as the environmental circumstances in the workplace, while *Work Activities* describe the different kinds of labor done by a worker. The following *Work Characteristics* were described in the matrix: lighting, noise, temperature, hazardous substances, safety hazards, and objects (e.g., ladders, buckets). Furthermore, the following *Work Activities* were described in the matrix: type of manual work, type of cognitive work, type of activity, place of activity as well as obstacles caused by working. These descriptions received the time stamp “0” as they were present at the start of the recording. Every time a characteristic changed (including the removal of an object or the ceasing of an activity), it was added in a new column to the right of the previous description of the characteristic together with a timestamp. A systematic analysis of the video recording resulted in a matrix describing different aspects of the plastering work over the recording period and builds the basis for the risk assessment.

### Sampling Method, Video Recording, and Gear

A construction company in Germany, specialized in plastering, interior fittings, and drywall construction, agreed to participate in the pilot study. One worker gave their informed consent and was recorded for a 30 min period while plastering. The study was approved by the ethics committee of the University of Kaiserslautern.

**Figure 1:**
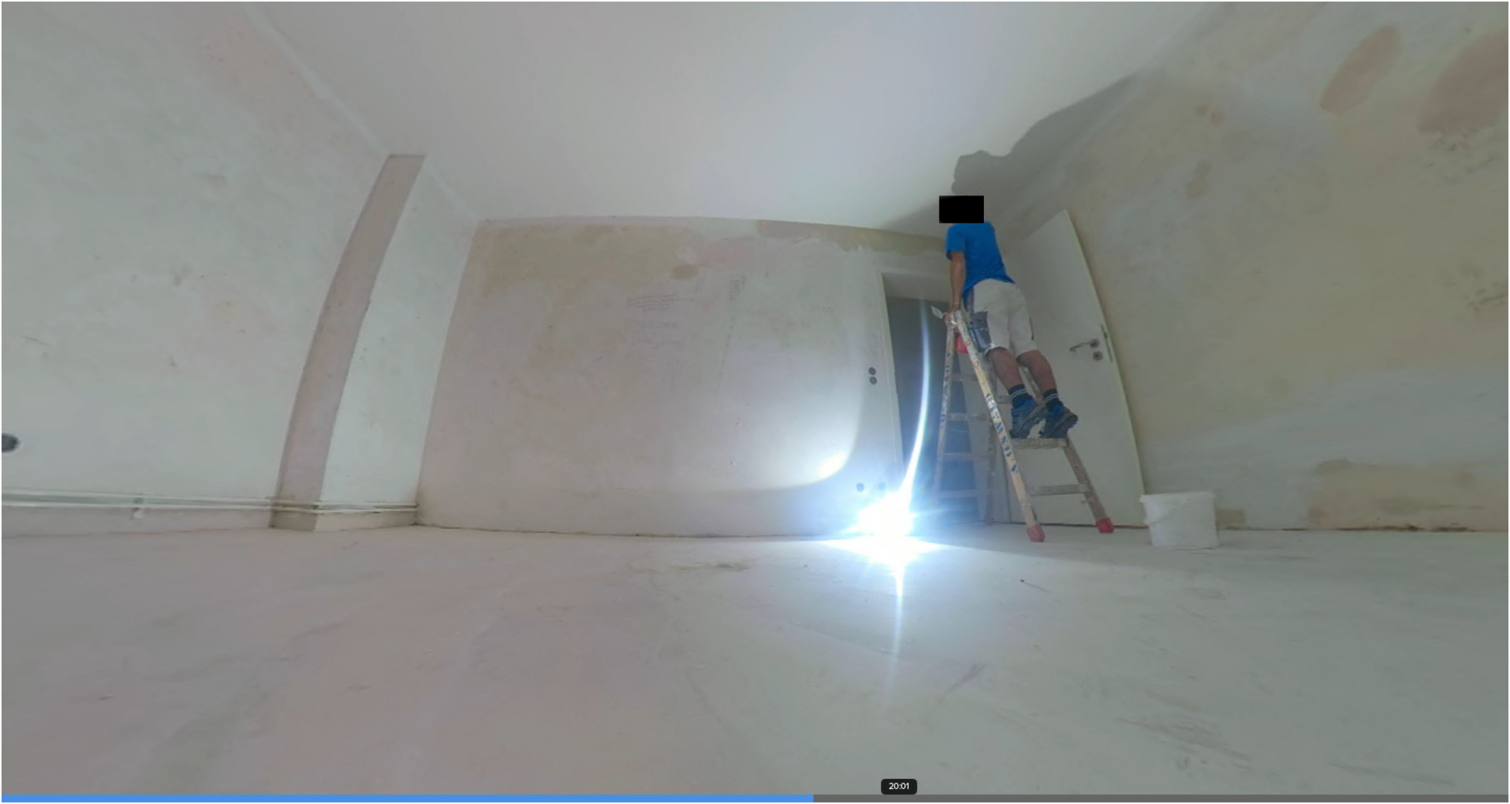
Image of 360 degree capture of plastering worker preparing walls and ceiling.

For the video recordings, we used a GoPro Fusion 360° camera. The resolution of the recordings was the 5.2 K mode with 30 frames/s. To create the 360° field of view, the camera uses two lenses with 190° field of view each. The camera was placed on the ground in the middle of the room to get the best possible view and to avoid hindering the construction worker during his work. The performed work was in an apartment and included cleaning of the walls, plastering the walls, and applying primer to the walls (see Figure 1). After finishing, the room was cleaned and the tools were stored by the worker before they turned off the camera. The face of the construction worker was masked after the recordings were done.

### Results of Analysis

The analysis of the video recording comprised the following: (i) the amount of time a worker was exposed to certain work environmental characteristics and the amount of time a worker carried out certain activities, (ii) how many objects and workers were present in a certain time period, and (iii) under what conditions workers carried out their activities. For the recording of a dry wall renovation, the following *Work Characteristics* and their duration were identified through observation of the video material (see Table 2):

- lighting: 8:39 min of half-dark conditions and 19:18 min of bright lighting,
- noise: 0:10 min of low-level noise,
- hazardous substances: dust at all times,
- safety hazards: possibilities included objects such as buckets, a ladder and renovation tools (i.e., trowels and plaster).

**Table 2:** Work characteristics and work activities.

**Work characteristics**	**Duration in mm:ss**
Hazardous substances	29:11
Noise	0:10
Lighting	8:39 (half-dark), 19:18 (bright)
**Work activities**	**Duration in mm:ss**
Lifting	0:32 (bucket), 0:44 (ladder)
Plastering	22:02
Forced posture	0:15

The following aspects of the *Work Activities* were identified: lifting and carrying the ladders and tools within the room as well as using them to renovate (manual work), focusing attention, speaking, and planning (cognitive work), plastering and cleaning (type of activity), room of an apartment (place of activity), having a bucket or a ladder standing in your way (obstacles).

Further, the matrix of the video recordings summary revealed 5:05 min in which two objects were present and 0:06 min in which three objects were present. The conditions were 0:10 min under noise, 3:21 min standing on a ladder, 05:57 min doing manual and cognitive work simultaneously.

The characteristics and activities derived from the video recordings can be easily supplemented and reduced as wished by a company to match their workplace and environment.

**Figure 2:**
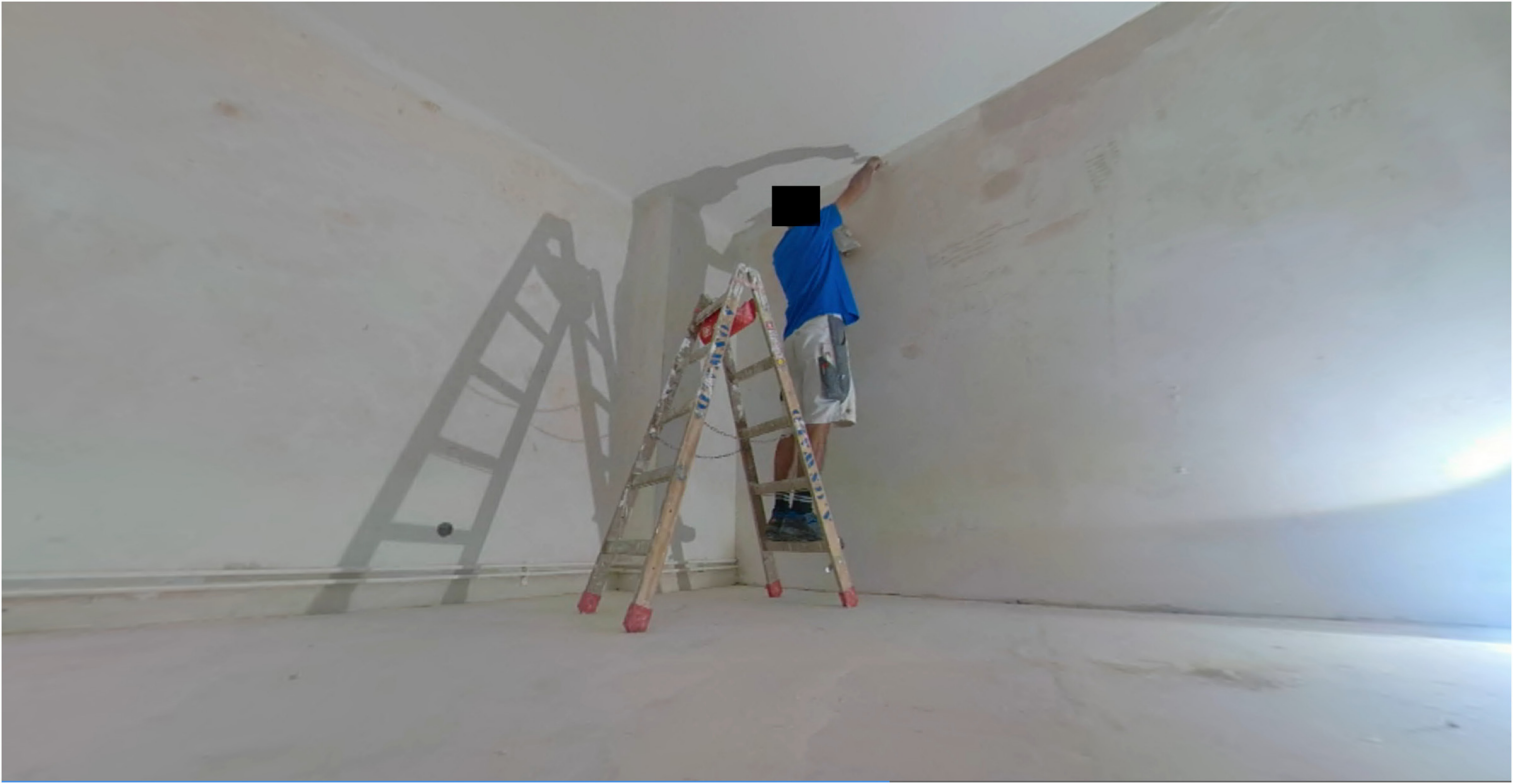
Plastering worker standing on a ladder in a potentially hazardous manner.

After analyzing the video material, the developed matrix is available for risk assessment at work. Comprehensive risk assessments often employ a risk assessment technique. Examples for such techniques include observational ergonomic methods or the task demand assessment (TDA), a new technique for measuring the safety risk of construction activities. In the TDA, you observe parameters that can affect the potential for accidents by quantifying “task demand” of actual operations based on characteristics of the activity independently of the workers' capabilities (30). Typically, the values for task demand range from 0 (no demand) to 9 (very high task demand) per task, the overall task demand of the work is then the sum of all task demands. Strictly for testing purposes, an analysis of the tasks and risk factors was done using the TDA based on the developed matrix. Accordingly, we divided the work of plastering into three main task demand factors: lifting, the possibility of falling from the ladder, and the posture while on the ladder (i.e., standing sideways, back toward the ladder, head toward the ladder). The task demand value of lifting was dependent on the size and weight of the lifted object, with the value of lifting a bucket being 1 (very low) and lifting the ladder being 3 (low). The maximum elevation while the ladder was 6 feet. Depending on the rung of the ladder the worker stood on, the task demand value of falling ranged from 3 (low) to 6 (moderate). Posture-wise, the value ranged from 3 to 6, with standing with your back to the ladder on the ladder being the moderate task demand (see Figure 2).

The matrix also contains enough suitable dimensions to be compatible with existing methods such as the REBA (31) and the Belastungs-Dokumentations-System [BDS, (32)]. Such dimensions include work posture, strain on the visual and auditory systems, psychological monotony, and stress.

To further analyze how the aforementioned *Work Characteristics* (see Table 2) contribute to risk and act as risk factors, an exposure assessment is needed. Without using sensors, the matrix can only be used to analyze whether one of those characteristics is generally present and the amount of time it is present during a recording. This also means that for example the lighting can only be analyzed qualitatively and not quantitatively. To get the most out of the matrix and add more quantitative measures, sensor data on exposure to those characteristics similar to VEM/PIMEX is needed.

## Discussion

While similar work has been done (33, 34), the new method we developed to measure observational characteristics in an objective, quantifiable manner is more suitable for a complete case-by-case risk assessment. Our results indicate that using a 360° camera and analyzing the video recordings can be an easy to implement method that (i) avoids traveling or entering the workplace directly, (ii) provides necessary information to make an observational risk assessment, (iii) can be enhanced by using sensors and similar equipment to provide quantitative exposure data, and (iv) allows a detailed description of the workplace. The video recordings save all relevant information in an objective, quantifiable manner and avoids the problem of a natural observer possibly missing important details. Our results also indicate that the observational matrix is easy to complete and new codings for workplace characteristics can be added at any time, meaning that the method is easily transferable to different workplace scenarios.

### Practical Considerations and Significance

Accordingly, employing such a systematic risk assessment can identify important triggers for health care risks and advise relevant stakeholder groups on when preventative measures are necessary.

Systematic risk assessments are a necessary but not easily accomplished process in every company. The aspect that makes it particularly challenging is the complexity of the work environment. A paper by Dollard et al. (35) compared 35 national systems for risk assessment at work. They recommend that systems should be flexible enough to identify and assess emerging risk factors such as emotional demands, workplace harassment and violence, exposure to acute stressors, and positive psychological states and further recommend that stakeholders should cooperate with international systems operators to work toward an international observation system (35). To meet the demands of a comprehensive risk assessment, the greatest advantage is advancements in technology which allow for even further possibilities than we have proposed. Risk assessment systems or tools might also take multimodal input (i.e., auditory, visual, physiological) through sensor systems built specifically for risk assessment (36). The information from these systems could then be added to the *Work Characteristics* matrix derived from the video recordings.

The data could then be visualized in a simple and comprehensive way and hard to analyze areas in the data can be marked so that an expert can then decide if a hazard is truly present. This could be done using a semi-automated software tool to minimize observer errors and would thus lighten the workload of occupational physicians and risk assessors who would then only need to look into data deemed hard to classify by the software.

### Conceptual or Methodological Constraints

The method we developed for occupational risk assessment has shown promising potential. However, it is only a first step toward a usable digital approach for the assessment of work hazards. There needs to be further research on interrater reliability concerning experts and non-experts as well as research on different trades to test if it holds up in other situations and in regards to objectivity. The authors are working on this research to further improvement the developed method. The method currently is seen as most beneficial for case-by-case risk assessments of construction work and similar industries where it is easy to implement. In its current form, the matrix is only using data acquired through observation, not accounting for exposure of hazardous substances and similar characteristics. While there is a possibility to use sensor data in conjunction with the matrix, this pilot study did not use them due to time and material constraints. Therefore, it still needs to be tested how well the matrix would work when including sensor data.

Using sensor data as well as just video recordings also brings about ethics concerns and thus constraints (37). An employee as well as an employer needs to agree to being recorded as well as agree to the further use of the data for analysis. The recorded data needs to be as anonymous as possible. Therefore, video recordings should mask any faces and company logos if the employer or employee so desires, at least before the analysis, better yet in automated form during recording. Our video recordings were masked before analysis by a research assistant which can be very time consuming.

### Lessons Learned for Future Consideration

The first step—implemented in this study—was to lay the groundwork for defining and implementing the characteristics and requirements for the *Work Characteristics* matrix. The next step will be to expand the components of the matrix (including the coding list) to be able to make more aspects of the observation quantifiable in an objective manner. A further challenge is then to implement automatic algorithms for the analysis of the video recordings so that the workplace characteristics will be automatically translated into matrices, also including sensor data of exposure to hazardous substances. After having met these challenges, future work can focus on the automatic generation of summary scores and creating algorithms for automated risk assessment based on different techniques such as the aforementioned Task Demand Assessment (30) or the Rapid Entire Body Assessment (15) for an available video recorded work sequence, similar to Padilla et al. (38). This will facilitate a complex but necessary process of risk assessment in every company that is required to be completed regularly by law.

The digital observational approach should also help to create a comprehensive knowledge base, curated by experts, of quantitative measures, workplace analysis according to physical attributes (e.g., exceeding certain exposure criteria such as high temperature, high humidity, or long/high exposure to dust) of the work area, and physiological and mental attributes of the worker. Such data can also be used to derive a comprehensive knowledge base on risk combinations and health outcomes at work. This type of information is a first step to foster preventative interventions to protect health in the workplace. Moreover, analysis of the 360° videos will help to evaluate in the short-run whether preventative measures are acceptable and suitable in specific scenarios.

A collaboration with experts in occupational hygiene and occupational safety is planned to further develop our matrix.

Overall, we see this method as a first step toward an automated video-based risk assessment tool, as previously mentioned. An automated risk assessment tool could help to make the risk assessment easier for employees as well as employers. We hope that the described method in this paper leads others to further develop and also implement it in an automated tool.

## Data Availability Statement

The original contributions presented in the study are included in the article/Supplementary Material, further inquiries can be directed to the corresponding author/s.

## Ethics Statement

The studies involving human participants were reviewed and approved by Ethics Committee of the Social Science Department (Ethikkommission des Fachbereichs Sozialwissenschaften), University of Kaiserslautern, Kaiserslautern, Germany. The patients/participants provided their written informed consent to participate in this study. Written informed consent was obtained from the individual(s) for the publication of any potentially identifiable images or data included in this article.

## Author Contributions

NB, FR, and JS contributed to conception and design of the study. NB and FR wrote the first draft of the manuscript. NB, FR, JS, and TL wrote sections of the manuscript. All authors contributed to manuscript revision, read, and approved the submitted version.

## Conflict of Interest

The authors declare that the research was conducted in the absence of any commercial or financial relationships that could be construed as a potential conflict of interest.

## Footnotes

^1^Arbeitsmedizinischer Dienst der BG BAU. *Betriebsärztlicher Gesundheitsbericht für Stuckateure und Verputzer*. BGBau.de. https://www.bgbau.de/fileadmin/Themen/Arbeitsschutz/Arbeitsmedizin_Vorsorge/Gesundheitsbericht-Stuckateure_und_Verputzer.pdf
